# A-Site Residues Move Independently from P-Site Residues in all-Atom Molecular Dynamics Simulations of the 70S Bacterial Ribosome

**DOI:** 10.1371/journal.pone.0029377

**Published:** 2012-01-03

**Authors:** Relly Brandman, Yigal Brandman, Vijay S. Pande

**Affiliations:** 1 Chemical and Systems Biology, Stanford University, Stanford, California, United States of America; 2 Chemistry, Stanford University, Stanford, California, United States of America; University of Akron, United States of America

## Abstract

The ribosome is a large macromolecular machine, and correlated motion between residues is necessary for coordinating function across multiple protein and RNA chains. We ran two all-atom, explicit solvent molecular dynamics simulations of the bacterial ribosome and calculated correlated motion between residue pairs by using mutual information. Because of the short timescales of our simulation (ns), we expect that dynamics are largely local fluctuations around the crystal structure. We hypothesize that residues that show coupled dynamics are functionally related, even on longer timescales. We validate our model by showing that crystallographic B-factors correlate well with the entropy calculated as part of our mutual information calculations. We reveal that A-site residues move relatively independently from P-site residues, effectively insulating A-site functions from P-site functions during translation.

## Introduction

The dynamic motion of macromolecular machines such as the ribosome is coordinated across multiple chains to accomplish complex, multi-step functions and elucidation of these motions is fundamental to understanding how these large machines work [1], [2], [3], [4], [5]. The ribosome translates mRNA into protein by coordinated dynamics of its hundreds of thousands of atoms in more than fifty RNA and protein chains. These chains are assembled into two subunits: the large subunit catalyzes peptide bond formation, and the small subunit decodes mRNA by facilitating the binding of the mRNA codon to the corresponding tRNA anticodon. Co-factors and GTP hydrolysis catalyze events in protein synthesis, however the ability of the ribosome to translate without GTP hydrolysis [6] and even without any cofactors [7], [8] suggests that functionally important dynamics are intrinsic to the ribosome itself. The molecular details of ribosomal translation are still an active area of research, and understanding ribosome dynamics is central to understanding how this robust molecular machine orchestrates protein synthesis.

Our current understanding of ribosome dynamics began largely by comparing EM and x-ray crystallography structures of the ribosome in various states (for example bound to co-factors or tRNA) [1], [2], [3], [4]. The ribosome translates proteins on the order of seconds (e.g. ∼20 amino acids per second in *Escherichia coli*). Large-scale dynamics such as motion in the GTPase association center [9] and L1 stalk [10] (large subunit protuberances at the tRNA entrance and exit sites, respectively) have been measured on similar timescales using methods such as single molecule fluorescence resonance energy transfer (smFRET) [10], [11], [12]. Thus enzymatic timescales are on the seconds timescales, and there is likely a broad range of timescales characterizing ribosome dynamics. Elucidating motions in the ribosome continues to be an active area of research by both experimental and computational methods.

Computational models of ribosome dynamics are challenging because of the ribosome's large size. Methods for modeling enzyme dynamics range from reduced complexity coarse-grained models to the all-atom detail of explicit solvent molecular dynamics simulations (MD) [13], [14], [15], [16], [17]. The more detail included in a computational model, the more computationally demanding the calculations are. Coarse-grained models are able to access biologically relevant timescales (e.g. peptide bond formation occurs on the seconds timescale), while more detailed models such as MD require decades of CPU years to reach tens of nanoseconds for the millions of atoms in a system as large as the ribosome. Multiple, independent models of ribosome dynamics on various timescales complement each other and fill in molecular-level spatial and temporal timescales that can be difficult to access experimentally.

Our goal is to find features in nanosecond length all-atom MD of ribosome dynamics that are biologically relevant. Correlated motions have been previously linked to enzyme function [18], including coarse-grained simulations of the ribosome on the seconds timescale [15]. Correlated motions in short timescale (relative to biological timescales) MD have been used to gain insight in other systems (e.g. [19]). We expect that the ns dynamics in our simulations are largely from thermal motions and local conformational changes around the crystal structure rather than large-scale conformational changes. We calculate correlated motion in order to group atoms with coupled dynamics together. We hypothesize that atoms with coupled dynamics are functionally related, and that these relationships are also relevant on longer timescales.

Here we present data from two all-atom MD trajectories of the complete (both subunits, 70S) bacterial *Thermus Thermophilus* bacterial ribosome [20] and calculate correlations in residue dynamics using mutual information. One trajectory is of the ribosome alone (53 ns), and the other trajectory contains the ribosome with mRNA and tRNA (32 ns). Because of the short timescale (compared to the timescale for translation) and the fact that the initial starting configuration reflects a stalled ribosome in the pre-translocation state, we do not expect to see motions corresponding to translation. We expect that motions in our simulation are largely thermal motions on the ns timescale and local conformational changes around the crystal structure, thus our conclusions can be likened to characterizing a structure based on how it vibrates. We describe correlated motions between residues in order to characterize groups of atoms that may function together as “parts” of the larger macromolecular machine, thus providing insight into ribosome dynamics. Correlated motions corroborate previously established “parts” such as the two symmetry-related regions in the active site [21] and the peptide exit tunnel constriction site formed by two proteins [22]. Residues in the large subunit along the path of tRNA translocation, including the GTPase association center and L1 stalk, move relatively independently from the majority of the ribosome, consistent with their previously established stochastic dynamics. These regions show coupled motion to A- and E-site tRNA, respectively. Finally, we show that residues coupled to A-site tRNA move independently from residues coupled to P-site tRNA.

## Methods

### Molecular Dynamics

#### 53 ns trajectory of 70S ribosome without tRNA and mRNA

The starting structure for molecular dynamics was the highest resolution 70S crystal structure at the time of initiating the study, the 2.8 Å 70S bacterial Thermus Thermophilus ribosome crystallized in complex with mRNA and tRNA (PDB IDs 2J02 and 2J03) [20]. mRNA and tRNA were removed to create the starting structure. Residues and atoms missing from the crystal structure were added using MODELLER [23]. The ribosome was modeled in explicit Tip3p water [24]. A 255×295×285 Å box was created to accommodate the ribosome +10 Å on all sides to ensure that the enzyme would not interact with itself in the periodic boundary conditions. 640,243 water molecules fit into this box. Ions were included in the simulation for neutralizing charge and maintaining conditions as close as possible to crystallographic conditions. The crystal structure included 638 Mg2+ ions and 2 Zn2+ ions, and the positions of these ions were used in the starting structure. 2,359 K+ ions were added in random positions to neutralize the charge. K+ was chosen because the crystallographic buffer conditions contained excess K+ (5∶1 K+∶Mg2+, and no Na+, a commonly used positive ion in molecular dynamics simulations). The total number of atoms was 2,169,658.

Molecular dynamics was performed using the Gromacs software package [25] running on 256 nodes on the BioX2 cluster at Stanford University. The Amber99p force field [26] was used. Energy minimization using the steepest descent algorithm was used. A 200 ps equilibration at constant pressure using the Berendsen barostat [27] coupling the system to a pressure bath at 1.0 atm was performed. Following equilibration, the simulations were kept at constant volume. Simulations were run at 300 K with 2 fs timesteps. Long-range electrostatic interactions were treated by using the particle-mesh Ewald method [28] with a real space electrostatic cutoff of 1.2 nm. The Lennard–Jones potential, describing the van der Walls interaction, was cut off at 1.0 nm. Hydrogen bonds were constrained using the LINCS algorithm [29]. Temperature was kept constant by coupling the system to a temperature bath (300 K) using the V-rescale algorithm [30].

#### 32 ns trajectory of 70S ribosome with tRNA and mRNA

In the simulation with tRNA and mRNA, the methods are the same as above except for the following changes. mRNA and the three tRNA chains were included in the simulation, with missing residues and atoms added using MODELLER [23]. The template for missing A-site tRNA was based on the E-site tRNA. 2609 K+ ions were added (more ions were needed due to the addition negative charge from the additional RNA nucleotides). The box size was 257×301×286 Å. There were 649595 water molecules and 2206135 total atoms.

### Mutual Information

The mutual information between every pair of backbone atoms was calculated as follows:
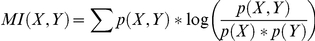
where x (or y) is the distance of atom x (or y) from the mean position of its trajectory over the interval t. Binning was at 1 Å resolution. Backbone atoms are C4′ (RNA) or Calpha (proteins). MI calculations where performed using the Matlab software package (The MathWorks, Inc). Normalized MI is calculated by dividing by the joint entropy (Shannon entropy), H(X,Y), as follows:
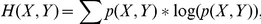


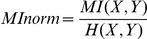
This method of normalization weighs low entropy residue pairs equally to high entropy residue pairs. Normalized MI can have a value between 1 (maximally correlated) and 0 (independent).

### Clustering

Kmedians clustering was performed using the Matlab software package (The MathWorks, Inc) using the pre-installed kmeans function with the “cityblock” parameter.

## Results

### Molecular Dynamic Simulations

We ran two all-atom MD trajectories of the complete (both large and small subunits, 70S) bacterial ribosome from *Thermus thermophilus*: one 53 ns trajectory of the 70S ribosome alone, and one 32 ns trajectory of the 70S ribosome with mRNA and tRNA (see Methods for details). Each simulation was run on 256 processors in parallel, with a total computational time of 50 CPU years. The (short) length of the trajectories reflect the large computational demand of the simulations, with each having more than 2 million atoms. The simulations were run as long as possible and limited by computational and time resources. The starting structure for both of these simulations was the 2.8 Å crystal structure of the ribosome in a pretranslocation state formed by binding of mRNA, deacylated initiator tRNA^fMet^ in the P-site, aminoacyl tRNA^Phe^ in the A-site, and the antibiotic parmomycin to increase the affinity of A-site tRNA and inhibit translocation [20] (PDB IDs 2J02 and 2J03). In this structure, non-cognate tRNA was bound at the E-site. We chose this structure because it was the highest resolution 70S ribosome crystal structure when we began our study. Except for residues missing from the A-site tRNA, missing residues were added using MODELLER [31]. Except for the anticodon stem loop, most of the A-site tRNA was missing from the crystal structure (most likely due to disorder caused by tRNA deacylation during crystallization [20]. Missing residues for the A-site tRNA were modeled by translating the coordinates of the (equivalent) E-site tRNA. The coordinates of both trajectories remained stable over time. Energy minimization and equilibration in the Amber99p force field resulted in a C_alpha_ rmsd shift of about 10 Å relative to the crystal structure for the trajectory with (10.4 Å) and without (11.2 Å) mRNA and tRNA. These equilibrated structures were the starting structures for the trajectories, and during the simulation the C_alpha_ coordinates only shifted 2 to 3 Å on average. In the trajectory without mRNA and tRNA, the ending structure had 9.5 Å C_alpha_ rmsd relative to the crystal structure. In the trajectory with mRNA and tRNA, the ending structure had 10.1 Å C_alpha_ rmsd relative to the crystal structure.

### Calculating Correlated Motion

#### DMA as a Reduced Representation of MD data

The large size of the ribosome (200,000+ atoms) renders previous methods for calculating MI infeasible and thus we simplify the data into a distance from a moving average (DMA) for each residue. Previous methods used raw MD data of dynamics in the form of the (x,y,z) coordinates of every atom over time [18]. A more recent implementation published as this manuscript was in preparation uses protein phi/psi angles [32]. For our simulations, the dynamics for each residue was represented as the distance between one backbone atom (C_alpha_ for protein, C4′ for RNA) and the average position of that backbone atom within a time window (DMA). DMA is one dimension (distance) over time rather than three dimensions (x,y,z coordinates) over time. This representation has the following properties: 1. MI between residue dynamics is direction independent, 2. MI between residue dynamics will be independent of the location of the residue relative to either the origin or to other atoms in the system, and 3. the reduced complexity is more computationally tractable than previous methods [18] and thus it is feasible to analyze large datasets. See methods and supporting information S1 for more details.

#### Calculating Correlated Motion: Mutual Information

Mutual Information (MI) is the most general form of calculating correlation, and has been shown to capture more biologically relevant dynamics than traditional methods of calculating correlations such as principal component analysis (PCA) [18], [32]. MI is derived from information theory and captures all correlations, including non-linear and higher-order correlations not captured by PCA. We calculated the MI in the DMA for all pairs of residues (the MI matrix). See methods for more details.

**Figure 1:**
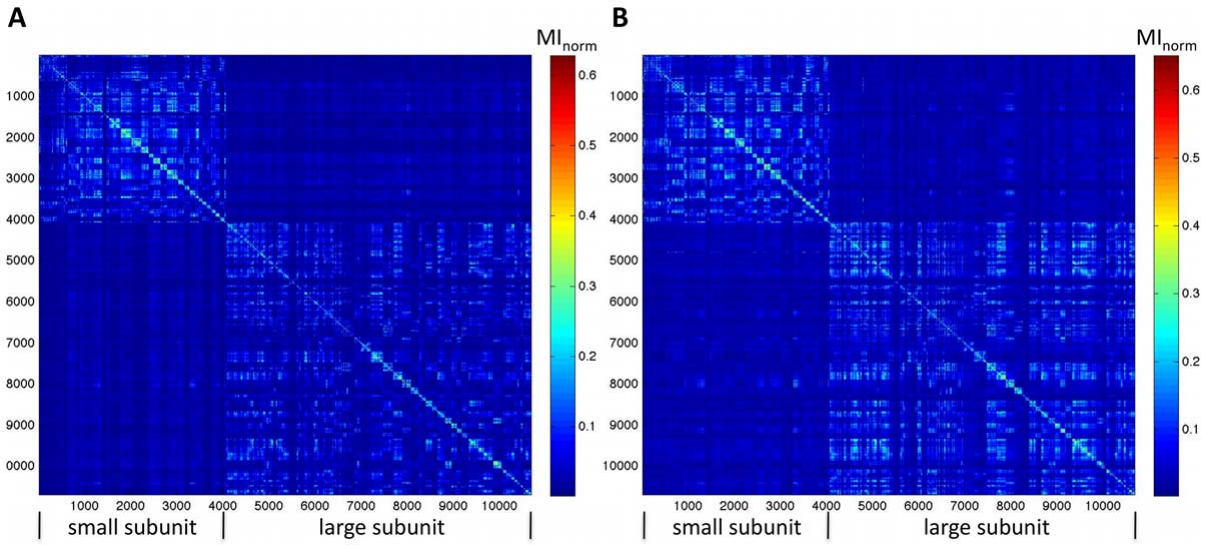
MI_norm_ in the dynamics between all pairs of residues in a 53 ns MD trajectory of the ribosome alone (A), and a 32 ns MD trajectory of the ribosome with mRNA and tRNA (B). High MI_norm_ indicates that two residues have coupled dynamics (red), and low MI_norm_ indicates that two residues move relatively independently from one another (blue). There is more coupling within a subunit than between, and within protein chains than within RNA chains. To increase the color contrast the MI_norm_ values along the diagonal (representing the MI between a residue and itself, by definition the highest MI for that residue) have been removed.

Figure 1 is the normalized MI (MI_norm_) matrix for the trajectory of the ribosome alone (Figure 1A) and the trajectory of the ribosome with tRNA and mRNA (Figure 1B). Each row and column in the symmetric matrix represents the MI_norm_ between that residue and all other residues. The higher the MI_norm_ between two variables, the more coupled the motion between two residues (high MI_norm_ is red and low MI_norm_ is blue). Conversely, low MI_norm_ indicates that two residues move relatively independently from one another. By definition, the highest MI is between a variable and itself, which results in a bright diagonal line through the matrix. To increase the color contrast when visualizing the matrices, the values along the diagonal are removed in all figures. The order of residues in these matrices mirrors the organization of chains in the pdb files: small subunit chains, then large subunit chains. Within the subunits, RNA chains are listed before protein chains. Table S1 in Supporting Information delineates the indices for each chain. Coupling within a subunit is greater than between subunits. Coupling within protein chains is greater than within RNA chains, consistent with the more compact globular structures of proteins relative to RNA.

#### Model Validation: Conformational Entropy correlates with Crystallographic B-factors

Correlation between residue root mean squared fluctuation (RMSF) and crystallographic B-factors (Debye-Waller temperature factors) is commonly used to validate computational models of protein dynamics such as MD [33]. B-factors capture positional uncertainty from protein mobility (signal) and model error and lattice defects (noise). Despite the noise, qualitative agreement with computational data is expected and correlations are still informative to assess. We compared simulation predictions of residue fluctuations with crystallographic B-factors to validate our simulations as well as the reduced complexity representation of residue dynamics as DMA.

**Figure 2:**
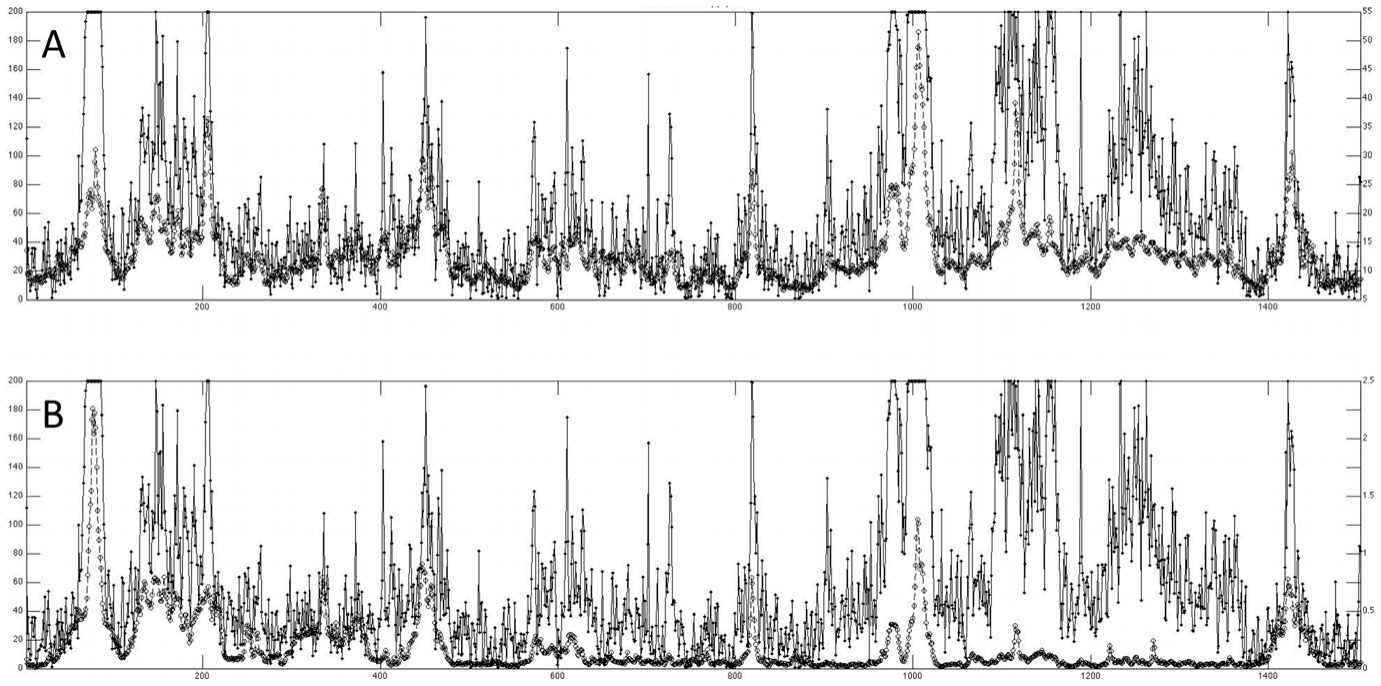
Experimental positional flexibility (crystallographic B-factors, closed circles and left axes in (A, B)) correlates with computational positional flexibility. (A) B-factors for C4′ atoms of each residue (closed circles) are compared with entropy in DMA (open circles) for the 16S RNA chain, the largest RNA chain in the small subunit. The correlation coefficient is 0.7. (B) B-factors for C4′ atoms of each residue (closed circles) are compared with the root mean squared fluctuation (RMSF, open circles) for the 16S RNA chain. The correlation coefficient is 0.5. B-factors are taken from pdb ID 2J02 [20]. Computational data is from the 53 ns trajectory of the ribosome alone.

We calculated the correlation between two measurements of positional fluctuations and crystallographic B-factors for the largest ribosome chains. Both RMSF and DMA entropy (a measurement of positional fluctuation based on DMA, see methods for details) correlate with crystallographic B-factors, and DMA entropy generally correlates better than RMSF. Figure 2 compares B-factors with DMA entropy (Figure 2A, correlation coeff) and RMSF (Figure 2B) in the 53 ns trajectory of the ribosome alone for the largest RNA chain in the small subunit, 16S. Table 1 summarizes the correlation coefficients for other ribosome chains and both ribosome trajectories. DMA entropy generally correlates better with B-factors than RMSF.

**Table 1:** Correlation coefficients between experimental positional flexibility (crystallographic B-factors) and two computational indicators of positional flexibility.

	53 ns trajectory ribosome alone		32 ns trajectory ribosome + mRNA + tRNA	
ribosome chain	correlation between CA B-factors and RMSD*	correlation between CA B-factors and entropy in DMA**	correlation between CA B-factors and RMSD*	correlation between CA B-factors and entropy in DMA**
16S RNA	0.5	0.7	0.6	0.7
23S RNA	0.6	0.7	0.4	0.6
S2 protein	0.1	0.5	0.6	0.4
L4 protein	0.6	0.7	0.6	0.6
L22	0.7	0.4	0.4	0.4

*to compare with B-factor units of Å^2^, we use RMSD^2^ because RMSD is in units of Å.

**to compare with B-factor units of Å^2^, we use e^entropy^ because entropy is on a logarithmic scale.

#### Mutual information in the active site: A- and P- loops move independently

**Figure 3:**
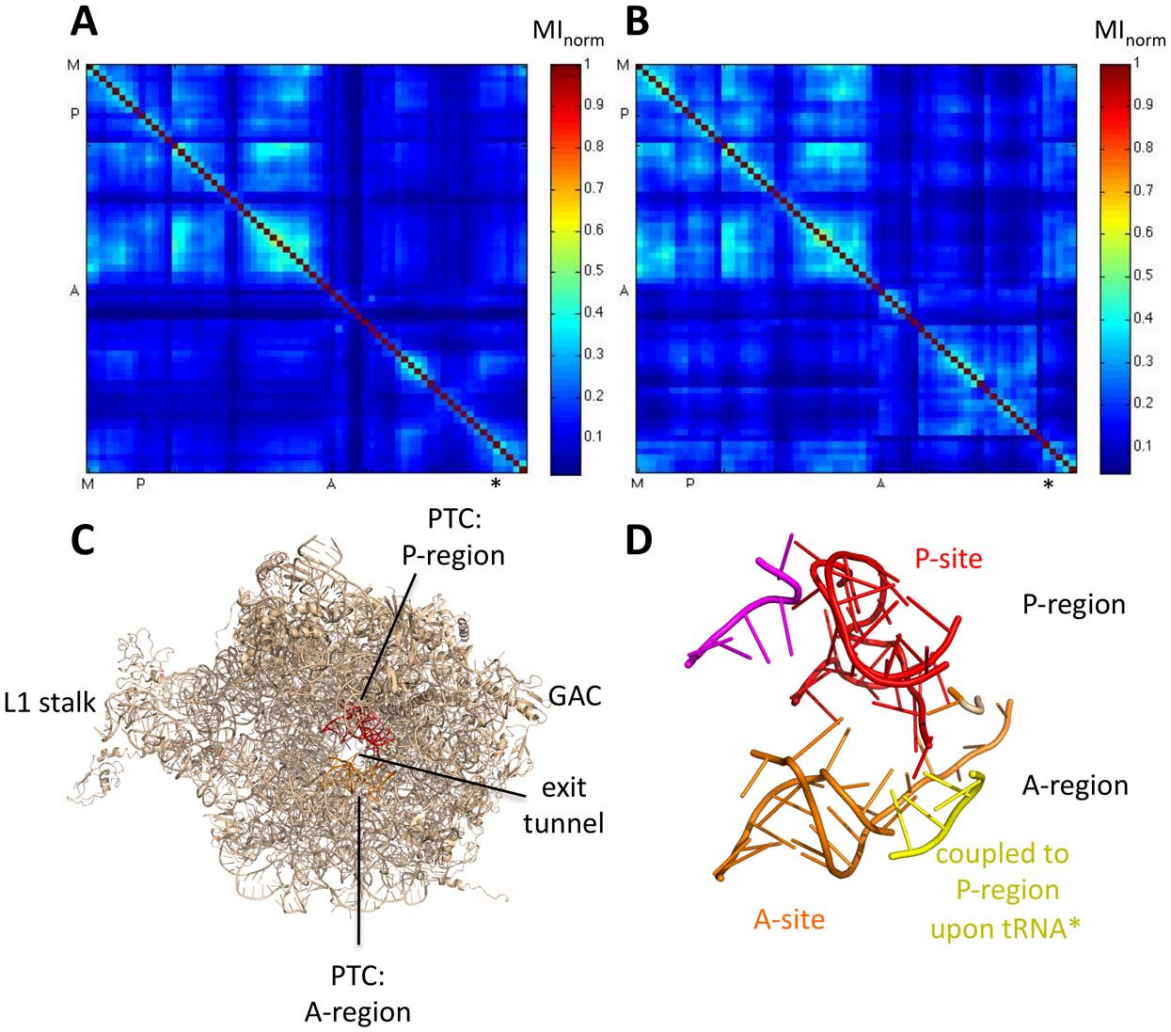
MI_norm_ in the catalytic site, the peptidyltransferase center (PTC), reveals a coupled P-region that moves independently from an A-region. MI_norm_ between all residues in the PTC for the trajectory of the ribosome alone (A) and the ribosome with mRNA and tRNA (B). 23S residues 2056–2063, 2250–2254, 2447–2454, and 2492–2507* have coupled dynamics in both trajectories (P-region, colored red in (C) and (D)). This group of residues moves relatively independently from the second group of atoms, 23S residues 2552–2556, 2573, 2582–2591 and 2601–2614 (A-region, colored orange in (C)). These two groups correspond to the two symmetry-related regions of the PTC [21]. Two main differences are apparent in the dynamics with (A) and without (B) mRNA and tRNA: 1. dynamics in the A-loop become coupled in the presence of tRNA, and 2. residues 2610–2614 (colored yellow in (D)) become more coupled to the P-region. *E.coli numbering throughout.

The active-site of the ribosome is the peptidyltransferase center (PTC) and is composed entirely of RNA nucleotides from the 23S chain of the large subunit. The PTC catalyzes peptide bond formation between the nascent peptide chain and the incoming amino acid. The PTC is located at the entrance to the 100 Å-long peptide exit tunnel, a cavity through which the nascent peptide grows and eventually exits the ribosome. The PTC binds to A- and P-site tRNA through the A- and P-loops, respectively (Figure 3). Each loop is on one of two symmetry-related regions that are hypothesized to have arisen from the relic of a dimeric proto-ribosome [21]. The two symmetry-related regions were discovered by observing structural symmetry conserved across ribosome crystal structures from multiple species [34].

MI_norm_ in PTC dynamics reveals a P-region and an A-region that move independently from one another, and these two regions correspond to the previously described symmetry-related regions (Figure 3). MI_norm_ between all residues in the PTC is shown for the ribosome alone (Figure 3A) and the ribosome with mRNA and tRNA (Figure 3B). P-region residues (23S residues 2056–2063, 2250–2254, 2447–2454, and 2492–2507, *E. coli* numbering used throughout) have coupled dynamics in both trajectories (colored red in Figure 3C). P-region residues move independently from the A-region residues (23S residues 2552–2556, 2573, 2582–2591 and 2601–2614 (colored orange in Figure 3C).

Residues in the A-loop, which bind to A-site tRNA, are uncoupled in the trajectory of the ribosome alone and are coupled in the presence of mRNA and tRNA. The A-loop moves independently from the other residues in the PTC both in the absence and presence of tRNA. These data suggest the role of dynamics in A-loop function: A-loop residues can interact with tRNA residues without influencing motion in other regions of the active site.

*Residues in the A-site, interface with the small subunit, and protuberances move independently from the rest of the large subunit* Residues can be clustered according to their MI with other residues in the simulation to reveal groups of residues with similar dynamics. We clustered the residues in the large subunit using the k-means algorithm (see methods for details). The algorithm clusters residues with similar MI_norm_ patterns, specifically the MI_norm_ between the residue of interest and all other residues in the large subunit (i.e. a row in the MI_norm_ matrix).

**Figure 4:**
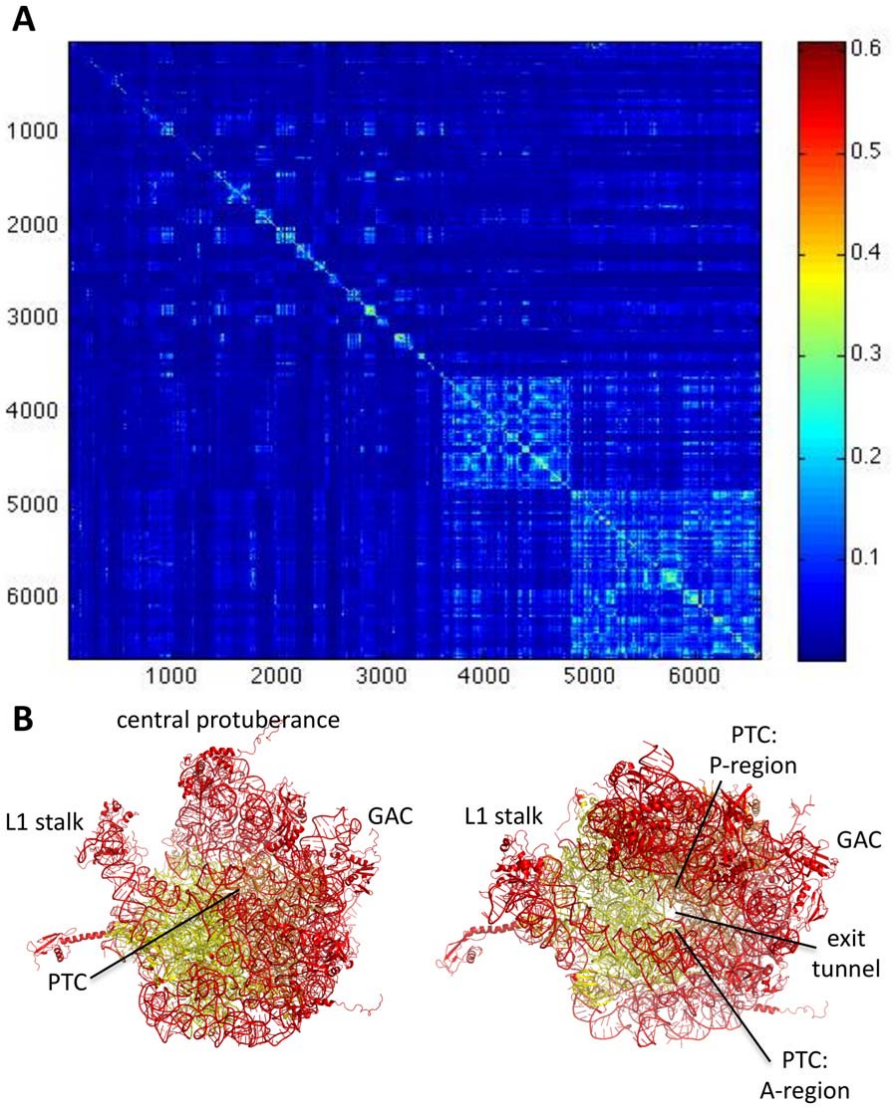
K-means clustering of large subunit residues into three clusters based on mutual information in dynamics. (A) Mutual information between all residue pairs in the large subunit. Residues are ordered according to cluster. Cluster 1 residues (colored red in (B)) move relatively independently from the rest of the subunit (low MI_norm_). Cluster 2 (color orange in (B)) and cluster 3 (colored yellow in (B)) residues show coupled motion between residues in the same cluster (high MI). Residues in cluster 2 and cluster 3 are more coupled to each other than they are to cluster 1. (B) Residues in each cluster are shown on the crystal structure of the large subunit. Cluster 1 residues (colored red) are the three protuberances that mark the tRNA translocation path and the residues that interact with the small subunit. Cluster 1 includes the A-loop residues that bind to A-site tRNA. Cluster 2 (colored orange) and cluster 3 (colored yellow) residues make up of the center of the subunit, including residues that surround the peptide exit tunnel. Cluster 2 residues include the P-loop residues that bind to P-site tRNA. Data based on the trajectory of the ribosome alone.

K-means clustering of the large subunit into three clusters reveals that residues in the A-site, at the interface with the small subunit, and along the path of tRNA move independently from the rest of the large subunit. Figure 4A shows the MI matrix reorganized according to clusters, and Figure 4B shows the same clusters on the crystal structure. Cluster 1 (red in Figure 4B) residues include residues in the A-site, at the interface with the small subunit, and in the three protuberances. Residues within this cluster are less coupled to one another than residues within clusters 2 and 3. Rather than moving collective as “one part”, cluster 1 is characterized by smaller groups of residues that move together, and these smaller groups move relatively independently from one another. Clusters 2 and 3 make up the center of the large subunit, including the peptide exit tunnel. Cluster 2 contains the P-site.

**Figure 5:**
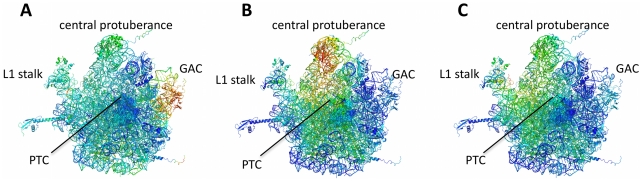
MI_norm_ between tRNA and residues in the large subunit. High MI_norm_ is indicated in red, and low MI_norm_ is indicated in blue. The PTC is depicted as spheres. (A) A-site tRNA dynamics are correlated to residues in the GTPase association center. (B) P-site tRNA dynamics are correlated to residues in the central protuberance, the P-site region of the PTC, and the peptide exit tunnel. (C) E-site tRNA dynamics have the least correlated dynamics to the active site. Residues in the large subunit that are most correlated to E-site tRNA are in the L1 stalk, central protuberance and along the peptide exit tunnel.

*Residues coupled to A-site tRNA are at the GTPase association center, and residues coupled to P-site tRNA are in the active site and exit tunnel* Mutual information between each of the three tRNAs and residues in the large subunit is calculated. Each tRNA has a different subset of residues with correlated dynamics, outlined in Figure 5. A-site tRNA is most coupled to residues in the GTPase association center (Figure 5A). P-site tRNA is most coupled to residues in the central protuberance, P-region of the active site, and along the exit tunnel (Figure 5B). E-site tRNA has the least coupled dynamics to the large subunit of all three tRNAs. E-site tRNA is most coupled to residues in the central protuberance and along the exit tunnel, however to a lesser extent than P-site tRNA (Figure 5C).

## Discussion

Correlated motion between residues is essential for enzyme functions such as interaction with ligands, catalysis, and allostery [18], [35], [36]. Correlated motion in the ribosome has been characterized previously by coarse-grained simulations modeling global motions related to translation [15]. Here we analyze correlated motion in all-atom MD simulations of the 70S bacterial ribosome with (one 53 ns trajectory) and without (one 32 ns trajectory) tRNA. The all-atom detail in these simulations comes at the expense of timescale: MD simulations are not computationally tractable for the seconds timescale of translation. Dynamics in these simulations are likely from thermal fluctuations and local conformational changes around the crystal structure. We hypothesize that correlated motion in these small timescales reveal groups of residues, or “parts”, with coupled motion that may function together on larger timescales.

We use MI to calculate correlation because it captures both linear and non-linear correlations, and we validate this method by comparing the crystallographic B-factors (an experimental metric of positional variability) with DMA entropy (a computational metric of positional variability). Crystallographic B-factors correlate well with DMA entropy (Figure 2). Correlation is stronger between B-factors and DMA entropy than between B-factors and positional root mean square fluctuation, a more standard metric of positional variability that is commonly compared to B-factors to validate molecular dynamics simulations.

Translation of mRNA into protein by the ribosome is an inherently dynamic process. tRNA is repeatedly shuttled from the aminoacyl (A) to the peptidyl (P) to the exit (E) sites located at the interface between the large and small subunit. Large conformational changes accompany translation. One such motion occurs at the protuberances at the tRNA entrance (GTPase association center) and exit (L1 stalk) sites that interact with incoming and outgoing tRNA, respectively. Previous simulations of correlated motion in the ribosome use low-resolution models to simulate longer timescales than the all-atom simulations described here, and captured these large scale motions [15], [37]. Despite short timescales, our simulations corroborate these previous simulations by showing that these protuberances are able to move independently from the rest of the enzyme (in other words, conformational changes in the protuberances do not disturb the conformation of the rest of the enzyme). In addition, we see correlated motion between the protuberances and their respective tRNA, which corroborates previous low resolution models [37] and is consistent with their function in translocation. Groups of residues that have correlated motion in our dynamics are also consistent with other previously described functional units in the ribosome, for example the ‘head’ region in the small subunit that is known to undergo rotation [38], [39], [40], and the A- and P- sites in the catalytic center of the ribosome, the PTC [21]. Thus we validate our models by showing that they are consistent with and complement previous data.

Our models suggest that residues involved in P-site functions move independently from residues in A-site functions. For example, residues in the PTC active site form two distinct groups based on MI in their dynamics: A-site residues, and P-site residues. These two groups move independently despite their close spatial proximity (Figure 3). Residues that are coupled to A-site and P-site tRNA (Figure 5) also move independently from one another. This data suggests that motion in the A-site is insulated from motion in the P-site, and vice versa. This observation is consistent with evidence that A-site tRNAs sample the mRNA codon multiple times before selection with little associated motion in the P-site tRNA [41]. Our results suggest that residues with coupled motion on short timescales are functionally related, adding to the growing literature suggesting that ribosome dynamics are critical to function.

## Supporting Information

Supporting Information S1 **Calculating MI with several parameters for distance from a moving average (DMA).** A range of parameters lead to the same patterns in correlated motion.
(DOCX)

Table S1 **Explanation of indices used in the MI_norm_ matrices.**
(DOCX)
