# Supplementary material for: A-Site Residues Move Independently from P-Site Residues in all-Atom Molecular Dynamics Simulations of the 70S Bacterial Ribosome
Source: PLoS One. 2012 Jan 3;7(1):e29377. doi: 10.1371/journal.pone.0029377 (PMC3250440; doi:10.1371/journal.pone.0029377)
Supplement: Supporting Information S1 — Calculating MI with several parameters for distance from a moving average (DMA). A range of parameters lead to the same patterns in correlated motion. (DOCX) [file pone.0029377.s001.docx]

*Calculating MI with several parameters for distance from a moving average (DMA)* Optimization of the parameters used to calculate DMA depends on the types of insights of interest. For the purposes of this study, we wanted to capture general motions in the entire ribosome and we aimed to use parameters that seemed biologically reasonable. A time window size of 500ps and 1ns (MD data collected every 10ps) resulted in similar MI (-0.76 < difference < 0.5 bits). MI for the entire trajectories (53ns for ribosome alone, and 32ns for ribosome with mRNA and tRNA) was similar to trajectories of the same length (from 5 to 32 ns for both trajectories, +/- <0.6 bits). The data presented in this work uses a 500ps time window and the entire trajectories. Further studies could include other parameters, for example DMA of side chain atoms or time-staggered correlations.
