# Supplementary material for: A-Site Residues Move Independently from P-Site Residues in all-Atom Molecular Dynamics Simulations of the 70S Bacterial Ribosome
Source: PLoS One. 2012 Jan 3;7(1):e29377. doi: 10.1371/journal.pone.0029377 (PMC3250440; doi:10.1371/journal.pone.0029377)
Supplement: Table S1 — Explanation of indices used in the MInorm matrices. (DOCX) [file pone.0029377.s002.docx]

**Table S1: Indices Corresponding to Each Chain**

|  | **70S Alone** | **70S + mRNA + tRNA** |
| --- | --- | --- |
| **Chain** | **Index Number** | **Index Number** |
| 16S RNA | 1 | 1 |
| P-site tRNA | 1523 | N/A |
| E-site tRNA | 1600 | N/A |
| mRNA | 1676 | N/A |
| A-site tRNA | 1700 | N/A |
| protein S2 | 1776 | 1522 |
| protein S3 | 2032 | 1778 |
| protein S4 | 2271 | 2017 |
| protein S5 | 2480 | 2226 |
| protein S6 | 2642 | 2388 |
| protein S7 | 2743 | 2489 |
| protein S8 | 2899 | 2645 |
| protein S9 | 3037 | 2783 |
| protein S10 | 3165 | 2911 |
| protein S11 | 3270 | 3016 |
| protein S12 | 3399 | 3145 |
| protein S13 | 3534 | 3280 |
| protein S14 | 3660 | 3406 |
| protein S15 | 3721 | 3467 |
| protein S16 | 3810 | 3556 |
| protein S17 | 3898 | 3644 |
| protein S18 | 4003 | 3749 |
| protein S19 | 4091 | 3837 |
| protein S20 | 4184 | 3930 |
| protein THX | 4290 | 4036 |
| 23S RNA | 4317 | 4063 |
| 5S RNA | 7104 | 6850 |
| protein L1 | 7224 | 6970 |
| protein L2 | 7453 | 7199 |
| protein L3 | 7729 | 7475 |
| protein L4 | 7935 | 7681 |
| protein L5 | 8145 | 7891 |
| protein L6 | 8327 | 8073 |
| protein L9 | 8507 | 8253 |
| protein L13 | 8655 | 8401 |
| protein L14 | 8795 | 8541 |
| protein L15 | 8917 | 8663 |
| protein L16 | 9067 | 8813 |
| protein L17 | 9208 | 8954 |
| protein L18 | 9326 | 9072 |
| protein L19 | 9438 | 9184 |
| protein L20 | 9584 | 9330 |
| protein L21 | 9702 | 9448 |
| protein L22 | 9803 | 9549 |
| protein L23 | 9916 | 9662 |
| protein L24 | 10012 | 9758 |
| protein L25 | 10122 | 9868 |
| protein L27 | 10328 | 10074 |
| protein L28 | 10413 | 10159 |
| protein L29 | 10511 | 10257 |
| protein L30 | 10583 | 10329 |
| protein L31 | 10643 | 10389 |
| protein L32 | 10714 | 10460 |
| protein L33 | 10774 | 10520 |
| protein L34 | 10828 | 10574 |
| protein L35 | 10877 | 10623 |
